# A Case of Stomatitis Materna Cured by the Syrup of the Phosphates

**Published:** 1859-11

**Authors:** J. C. Reeve

**Affiliations:** Dayton, Ohio


### Art. IV.—*A Case of Stomatitis Materna cured by the Syrup of the Phosphates.* By J. C. Reeve, M.D., of Dayton, Ohio.

I am desirous of laying before the profession a case in which a new remedy had a most happy effect upon a disease which is acknowledged to be always difficult of cure, and which sometimes sets all remedies at defiance.

The patient was a lady of rather delicate constitution, but who had generally enjoyed good health. She gave birth to her second child in March, 1855, being then about twenty-five years of age, and four or five months afterwards nursing sore-mouth made its appearance, with which she had not been troubled after her previous confinement. I was first called to prescribe for this complaint in January, 1856, and found her presenting all the distinctive features of this singular disease. I do not deem it necessary to enter minutely into the particulars of the case; the symptoms and appearances of nursing sore-mouth have been many times laid before the profession by those who have had more experience with it. I may refer, however, to the Report upon the disease, by Dr. McGugin, of Keokuk, Iowa, in the Transactions of the American Medical Association for 1858, as containing a description of the disease with which the case under consideration agreed in almost every particular. I especially remarked the burning pain along the course of the œsophagus and in the stomach after taking food, when the disease was worse than usual, and the constant aggravation of all the symptoms which followed the use of any of the subacid fruits. The report above mentioned contains an account of some cases far more severe than the one under my care, yet I may safely say that this one was severe enough to destroy almost every comfort of my patient's life for a period of nearly three years; and, as remedy after remedy was tried in vain, no slight amount of despondency was added to exert its depressing influence upon the health.

The ordinary remedies for the disease were prescribed as soon as I took charge of the case; but they proving of no benefit, weaning the child was recommended, with a feeling of confidence that it would put a speedy termination to the disease. In this, however, I was disappointed; it continued as bad as ever, and, without detailing particulars, I may say that almost every remedy I ever had seen prescribed or recommended for the disease was administered, without any permanent benefit. Singly, or in combination, I gave chlorate of potassa, syrup of iodide of iron, sulphate of iron with extract of gentian, iodide of potassium, tannin, gallic acid, quinine, decoction of bark with aromatics, subnitrate of bismuth with chalk and morphine, malt liquors, and lime-water. Locally I used the solid nitrate of silver to the ulcers, lotions of the same substance of various degrees of strength, lotions of tannin, and of glycerin with borax. Occasionally the disease would improve for a period under the use of some of these remedies, and for a few weeks be tolerably well, but only to break out again as bad as ever. Good effect was always produced by the use of chlorate of potassa, and of iodide of potassium in bitter infasions, while the use of bitter ale and lime-water always had a beneficial effect upon the general health.

In the autumn of 1858 the family removed to a prairie region of the Northwest, and I had encouraged my patient and her friends to look forward to a change of air and scene, and an entirely different mode of life, as likely to effect for her what medicines seemingly could not. But all parties again suffered a disappointment; and, being in correspondence with the family, I heard frequent complaints that the mouth was even worse than ever. Early in the spring of this year I saw an excellent recovery from scrofulous ophthalmia follow the use of the syrup of the phosphates, or chemical food, which had been dispensed by mistake for the tr. cinchonæ ferratæ; and it immediately occurred to me that it was worthy of a trial in a case where blood-making remedies are so clearly indicated as in nursing sore-mouth. I wrote to the friends, recommending a trial with it; before hearing from them Dr. McGugin's report fell into my hands, and the success he had obtained in one case with the hypophosphates encouraged me to give a similar preparation a trial. I therefore sent out a bottle of Blair & Wyeth's preparation. The patient commenced taking it in June last, at which time her mouth was as sore, if not worse than ever; she persevered with it for five weeks, at the end of which time her mouth was entirely well, which it had not been before since 1855, and her general health was so much improved that she says "she had forgotten what it is to feel in good health." She has taken the remedy occasionally since July, whenever she felt the need of a tonic, but has had no return of the sore-mouth. Her husband writes, under a late date: "For the first time in nearly four years she has eaten with impunity subacid fruits, etc. A month's trial with our wild plums gives no return of sore-mouth, while tomatoes are no longer tabooed. I think her in better health than she has been since the child's birth."

This case is reported, not with the idea that a specific has been found, but with the hope that an addition has been made to our means of controlling a disease which sometimes proves intractable. It is a single case, and as such is only valuable as encouraging to those who have cases upon which they have exhausted remedies and patience, to make one more trial. I may remark, however, that the many remedies prescribed, and the commendable perseverance of the patient in giving each not only a fair trial, but repeated ones, makes this case more than ordinarily valuable in establishing the fact of a cure by the medicine finally used.

